# Information Diffusion and Social Norms Are Associated with Infant and Young Child Feeding Practices in Bangladesh

**DOI:** 10.1093/jn/nxz167

**Published:** 2019-08-08

**Authors:** Phuong H Nguyen, Edward A Frongillo, Sunny S Kim, Amanda A Zongrone, Amir Jilani, Lan Mai Tran, Tina Sanghvi, Purnima Menon

**Affiliations:** 1 Poverty, Health, and Nutrition Division, International Food Policy Research Institute (IFPRI), Washington, DC, USA; 2 FHI 360, Washington, DC, USA; 3 University of South Carolina, Columbia, SC, USA

**Keywords:** breastfeeding, information diffusion, infant and young child feeding, social norms, Bangladesh

## Abstract

**Background:**

Interaction within mothers’ social networks can theoretically diffuse messages from interventions and campaigns into norms and practices for infant and young child feeding (IYCF).

**Objectives:**

We hypothesized that mothers’ social networks, diffusion of information, and social norms differed in intensive [intensive interpersonal counseling (IPC), community mobilization (CM), and mass media (MM)] compared with nonintensive (standard IPC and less-intensive CM and MM) intervention areas, were associated with IYCF practices, and partly explained practice improvement.

**Methods:**

We conducted household surveys at endline in 2014 and follow-up in 2016 (*n* = ∼2000 each round). We used multiple regression to test differences and changes in networks, diffusion, and norms within intervention areas. We analyzed paths from intervention exposure to IYCF practices through networks, diffusion, and norms.

**Results:**

Mothers’ networks were larger in intensive than in nonintensive areas in 2014 and increased in both areas over time [25–38 percentage points (pp)]. The prevalence of receipt of IYCF information was high, with no changes over time in intensive areas but an increase in nonintensive areas (8–16 pp). In both areas, more family members and health workers provided IYCF information over time. Sharing of information increased 17–23 pp in intensive and 11–41 pp in nonintensive areas over time. Perceived descriptive norms improved 8–16 pp in intensive and 17–28 pp in nonintensive areas. Perceived injunctive norms were high in both areas. Breastfeeding practices were associated with networks, diffusion, and norms (OR: 1.6–4.4 times larger comparing highest with lowest quartile). Minimum dietary diversity was associated with larger networks and diffusion (OR: 1.5–2.2) but not with social norms. Indirect paths from intervention exposure to practices explained 34–78% of total effects.

**Conclusions:**

Diffusion of IYCF information through social networks, reinforced by positive social norms for messages promoted over time, will contribute to positive changes in IYCF practices that may be achieved and sustained through large-scale social and behavior change interventions. This trial was registered at clinicaltrials.gov as NCT0274084.

## Introduction

Despite the critical role of optimal infant and young child feeding (IYCF) practices in improving child health, growth, and development (1), optimal practices have been adopted slowly and their adoption process is poorly understood. Social networks play a role in diffusing information and shifting social norms in the adoption of agricultural technology (2), use of family planning methods (3), success of education, health, and nutrition programs (4), and causes of childhood diarrhea and hygiene practices (5), but evidence for social networks (and norms) in the context of diffusion theory may not be explicitly detailed in the adoption of IYCF practices. A recent systematic review of 18 studies in 13 countries showed that mass media (MM) and nutrition education interventions have impacts on IYCF practices, but only a few studies used a formal theory; less than half included psychosocial factors related to IYCF among caregivers and few studies included a MM approach (6).

Practices of individuals are influenced by descriptive social norms (which refer to beliefs about the prevalence of a specific behavior, i.e., what other people do) and injunctive social norms (which refer to beliefs regarding the degree of approval of specific behaviors, i.e., what other people think) (7, 8). Beliefs about what is normative are influenced by individuals within the primary caregiver's social network such as other mothers, husbands, mothers-in-law, grandparents, other family members or neighbors within the community, and healthcare providers (9).

Social networks play a key role in diffusion of knowledge and practices among individuals (10, 11) with varying thresholds for adoption, that is, those with a low threshold adopt innovations even when few in the group have adopted, and those with a high threshold wait to adopt new innovations after many have decided to adopt (12). Information diffusion, which involves dispersal or spreading through information exchange, takes place among these early to late adopters within the social network until saturation is achieved or the information is abandoned. Social norms are established when enough people in a social network adopt a particular belief, practice, or technology, even those with a high threshold for adoption (12). Thus, social networks, diffusion of information, and social norms are highly interrelated and may influence the transformation of information into practice. Information from interventions and campaigns is received through social networks, within which individuals exchange messages with others who may influence interpretation and reinterpretation of the messages (12). Interaction among individuals within social networks can contribute to spreading information and translating messages into norms (13, 14).

The evidence on changing nutrition-related social norms through the diffusion of information is limited. A recent study in Bangladesh found that nonparticipant neighbors of households that participated in a nutrition education intervention demonstrated better IYCF knowledge and practices than nonparticipant neighbors of households that received cash or food alone, which may be caused by spillover effects from informal mother-to-mother interactions and from community meetings in which influential community members exchange information (15). In Burkina Faso, the size of the household's social network and the strength of the household's influence within its network had major effects on the mother's knowledge about both breastfeeding and complementary feeding (i.e., appropriate age of introduction of foods and feeding vitamin A–rich foods) (16).

**FIGURE 1:**
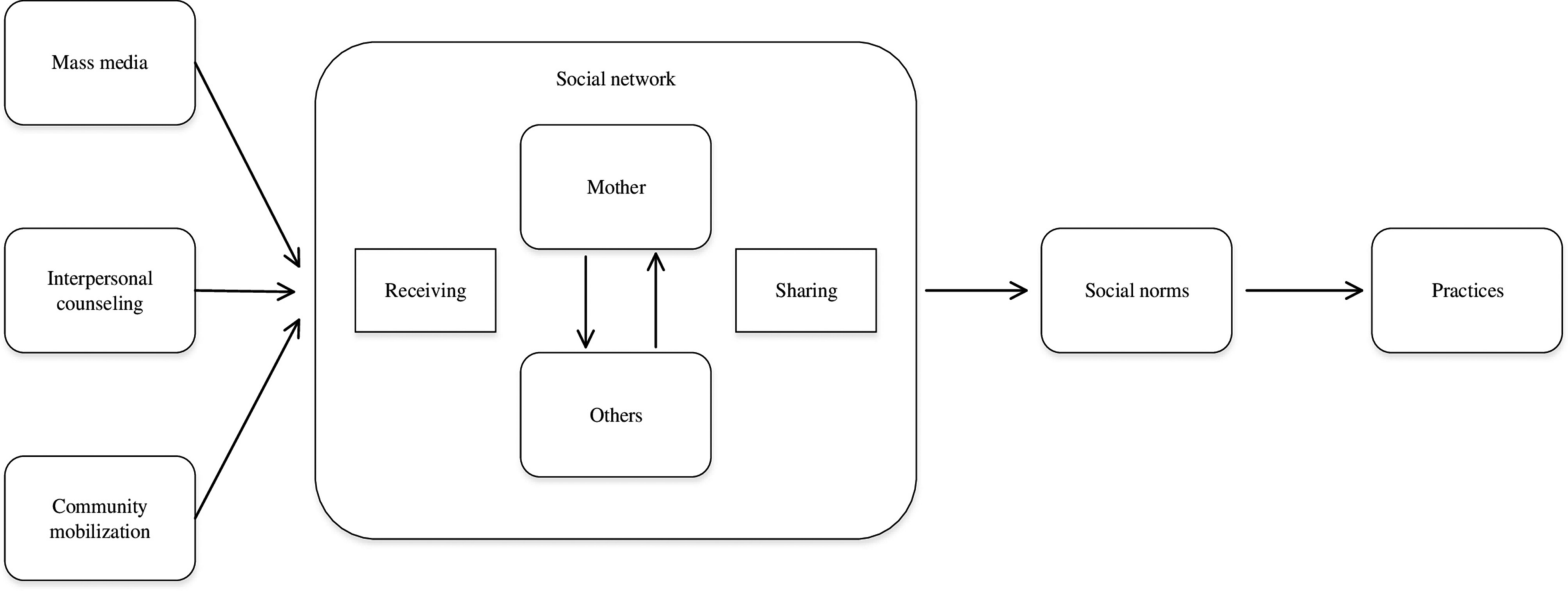
Conceptual framework for the relations between messages from interventions, social networks, norms, and practices.

In Bangladesh, Alive & Thrive (A&T), a multiyear initiative to improve IYCF practices (17), implemented intensive social and behavior change communication interventions through multiple channels from 2009 to 2014: interpersonal counseling (IPC), community mobilization (CM), MM campaigns, and policy advocacy to promote adequate IYCF practices and create an enabling environment for mothers and caregivers to adopt the recommended practices (18). The importance of social networks and diffusion of IYCF information to shift social norms related to IYCF was recognized as a necessary component of the intervention (19). We have previously shown that, by comparing randomly allocated intensive and nonintensive areas, the intensive intervention had substantial impact on improving breastfeeding (20) and complementary feeding practices (21), and these impacts were sustained 2 y after termination of initial external donor support (22). In this study, we aimed to advance understanding of how mothers’ social networks, diffusion of information, and social norms link receipt of intervention messages and IYCF practices by examining 3 hypotheses:

1. Mothers’ networks of known adopters, diffusion of IYCF information in terms of information received and shared by mothers, and social norms were *a*) larger in intensive compared with nonintensive areas at the endline when initial donor support terminated and *b*) changed more in the nonintensive than in the intensive areas between endline and 2-y follow-up.
2. Mothers’ networks, diffusion of IYCF information, and social norms were associated with IYCF practices.
3. Messages from the interventions were received by mothers’ networks within which diffusion of information occurred, leading to shifts in social norms and then practices (**Figure 1**).

## Methods

### Intervention description

Intervention implementation has been described in detail elsewhere (18, 20, 21). Briefly, 20 rural “upazilas” (subdistricts) with the Bangladesh Rural Advancement Committee's (BRAC's) essential healthcare program were randomly selected to receive A&T's intensive interventions (including intensified IPC, CM, and MM) or nonintensive interventions (standard IPC with less-intensive CM and MM). BRAC frontline workers and volunteers conducted routine home visits and provided standard information on IYCF practices in both intensive and nonintensive areas. In intensive areas, IPC was based on multiple age-targeted IYCF-focused visits to households with pregnant women and mothers of children aged ≤2 y by the frontline workers and volunteers, as well as home visits by a nutrition-focused frontline worker called Pushti Kormi (PK) who was an additional human resource, to provide more skilled support for IYCF practices. Performance-based incentives were given to the volunteers in the intensive areas.

The interventions were developed by incorporating ideas from behavioral (e.g., stages of behavioral change, reasoned actions, intentions and norms, interpersonal interactions, self-efficacy, and learning from role models) and community models (e.g., diffusion of innovation through social networks) (23). A theory of change was developed based on formative research about constraints on feeding practices and how to address them, specifying how impact was expected if specified strategies removed or mitigated constraints (24).

CM included awareness-building activities such as meetings and forums with husbands, religious leaders, health committee members, and other community leaders; and community theater shows focused on IYCF topics. The MM component, which consisted of 7 television spots containing emotionally appealing mini-dramas with various messages to spread the perception of recommended practices as normative, was televised nationally 12–24 times/d, 3 times/wk during prime viewing slots. The length of each TV spot and links to them are detailed in **Supplemental Table 1**. In intensive areas, added screening of the same TV spots along with a cartoon film on IYCF (“Meena”) was shown via community-based video shows in areas where TV reach was lower (“media dark” strategies), accompanied by community dialogue and quiz shows.

In total, from 2010 to 2014, an estimated 1.7 million mothers of children aged <2 y received IPC (21). In addition, BRAC held ∼5000 CM sessions reaching ∼1.6 million community members (25). The MM campaign reached an estimated 8.5 million mothers of children aged <2 y, in addition to fathers, grandmothers, health workers, doctors, and other members of society who influence feeding behaviors.

After 2014, BRAC's cadres of frontline health workers scaled up delivery of IYCF counseling and support services during home visits, antenatal and postnatal sessions, and health forums to >90% of the country. Although social mobilization activities focusing on IYCF had essentially diminished and the intensity of the MM campaign lapsed, the government of Bangladesh recently adopted the A&T MM materials and has been broadcasting the IYCF spots nationally since 2016.

### Data sources and study population

This study used data from a follow-up study to assess the sustained impact of A&T interventions (22), using a sampling design similar to that of the cluster-randomized impact evaluation (26). The household surveys were conducted at the intervention endline in 2014 and 2-y follow-up in 2016, consisting of interviews with mothers of children aged 0–23.9 mo (*n* = 2001 at endline and *n* = 2400 at follow-up).

### Measures

#### IYCF information diffusion

To assess the extent of IYCF information diffusion, we asked mothers in both intensive and nonintensive areas whether they had ever heard specific messages related to breastfeeding (such as early initiation of breastfeeding, feeding colostrum, no prelacteal feeding, exclusive breastfeeding, and no water or other liquids up to 6 mo) and complementary feeding (such as feeding mashed family foods, animal-source foods, and cooking children's food with oil). For each of the above items, mothers were asked about the sources of information (such as BRAC frontline health workers, other health workers, family members, community leaders, or MM), whether they shared the information, and with whom they shared the information (husbands, other family members, or others in their community). Mothers were able to select all that applied for each behavior. Each source of information or person with whom mothers shared was given a score of 1, and separate sums for information received or shared were created for the analyses.

#### Mothers’ social networks

To assess mothers’ networks of known adopters of specific IYCF behaviors, we asked mothers whether they knew other mothers who had adopted optimal IYCF practices in the community, then to specify those individuals they knew personally (name of each person, place they lived, and relation to mother). We set the denominator as all mothers, first to calculate the percentage of mothers with known adopters, then quantified the number of people in their social network; if they did not know adopters, then the number was zero.

#### Mothers’ perception of social norms

For social norms, we assessed mothers’ individual perceived social norms (both descriptive and injunctive) about IYCF practices. Information on mothers’ perceptions of social norms was obtained through the use of 2 types of questions: whether they believed most mothers in their community practiced optimal IYCF practices (descriptive norm, measured both at endline and follow-up), and whether they perceived that most people important to them approved or disapproved of practicing the recommended feeding behaviors (injunctive norm, measured at follow-up only). Each item was measured using a 5-point Likert scale in which women categorized the degree to which they agreed or disagreed with several statements. We constructed total scores for descriptive and injunctive norms by adding individual item scores.

### Data analyses

Descriptive analysis was used with the sample characteristics. Statistical testing for differences between intensive and nonintensive areas in a given survey period was performed with use of linear regression models (for continuous variables) and logistic regression models (for binomial variables), controlling for geographic clustering with district as a fixed effect and upazila as a random effect. A similar method was applied to test for changes over time within intensive and nonintensive areas. The sum scores of networks of known adopters (number of people known to have adopted IYCF practices), diffusion of IYCF information (received and shared information) and mothers’ perceived social norms (descriptive and injunctive norms) were categorized into quartiles, which were then used in a multiple regression analysis to test for the association with IYCF practices at follow-up. Path analyses were conducted to examine the paths from exposure to different A&T interventions on diffusion of information within mothers’ networks, leading to shifts in social norms and then practices. All models were adjusted for household socioeconomic status, maternal education, parity, child age, sex, and geographic clustering. Statistical analysis was performed with use of Stata version 15 software.

### Ethical clearance

Informed consent was obtained from the mothers of children aged 0–24 mo before their study participation. The research protocol received ethical clearance from the Bangladesh Medical Research Council and Institutional Review Board at the International Food Policy Research Institute. The evaluation is registered at clinicaltrials.gov as NCT02740842.

## Results

### Study sample characteristics

The mean age of surveyed mothers was 25 y, and the majority were housewives (**Table 1**). On average, mothers had 6 y of education; >10% had no schooling, and >80% did not complete high school. As intended by study design, about half of the sample of mothers had children aged <6 mo, and the other half had children aged 6–23.9 mo. There were no differences in maternal and household characteristics between intensive and nonintensive areas at endline and follow-up.

**TABLE 1:** Sample characteristics of household surveys, by intervention area and survey round^1^

	Endline 2014		Follow-up 2016	
Characteristics	Intensive (*n* = 1001)	Nonintensive (*n* = 1000)	Intensive (*n* = 1200)	Nonintensive (*n* = 1200)
Maternal characteristics				
Age of respondent mother, y	25.3 ± 5.4	24.7 ± 5.3	25.3 ± 5.3	24.9 ± 5.3
Years of schooling, y	5.8 ± 3.3	6.0 ± 3.3	6.3 ± 3.1	6.4 ± 3.2
Education level, %				
Never attended school	14.2	12.4	9.9	10.3
Primary school (grade 1–5)	30.5	30.9	29.7	28.7
Middle school (grade 6–9)	44.8	41.6	45.3	43.7
High school (grade 10–12) or higher	10.6	15.1	15.1	17.3
Occupation as housewife, %	76.2	84.1	92.1	94.4
Child characteristics				
Aged 0–5.9 mo, *n*	501	497	600	602
Aged 6–23.9 mo, *n*	500	503	600	598
Gender (female), %	51.8	50.1	48.8	51.5
Household characteristics				
Number of children < 5 y, *n*	1.3 ± 0.5	1.3 ± 0.5	1.4 ± 0.6	1.4 ± 0.6
Female household head, %	12.3	11.1	4.3	3.0
Ownership of house, %	96.2	94.5	99.6	99.1

1 Values are means ± SDs or percentages.

### Mother's social networks of adopters

**FIGURE 2:**
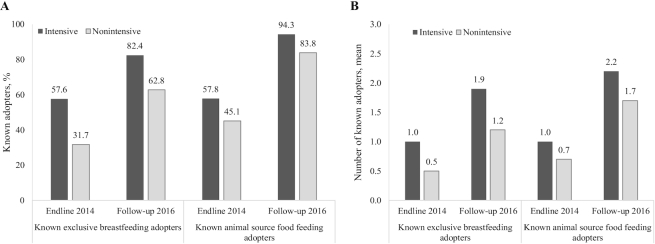
Mothers’ networks of adopters, by intervention area and survey round: (A) percentage of mothers who knew other mothers who had adopted optimal IYCF practices in the community and (B) size of mothers’ networks of known adopters of optimal IYCF practices. IYCF, infant and young child feeding.

The percentage of mothers who knew other mothers who had adopted optimal IYCF practices in the community was higher in intensive compared to nonintensive areas at endline 2014. Mothers’ social networks of adopters increased significantly in both areas over time (*P* < 0.01), with similar changes in both areas: 25–31 percentage point (pp) changes for exclusive breastfeeding (EBF) from birth until 6 mo and 36–38 pp changes for feeding animal-source foods to children aged 6–7 mo (**Figure 2**A). The mean number of known adopters of optimal IYCF practices also increased significantly (*P* < 0.05) and similarly in both areas over time (Figure 2B).

### Diffusion of IYCF information

Most mothers in intensive areas reported receiving IYCF messages at both endline and follow-up, with no significant difference over time (*P* >0.05) (**Table 2**). Although fewer mothers in nonintensive areas received these messages, the percentage of mothers who received the information increased significantly by 8–16 percentage points (pp) between endline and follow-up for various breastfeeding messages, 15 pp for feeding mashed family foods, and 14 pp for feeding animal-source foods (all *P* < 0.05). The changes over time were higher in nonintensive areas than in intensive areas for most messages (*P* < 0.05), except for the message about “cooking children's food with oil or mixing oil in children's food.” The main sources of IYCF information in intensive areas were BRAC frontline health workers (>80%) at both endline and follow-up, while other health workers and families were the main sources of information in nonintensive areas.

**TABLE 2:** Information received by mothers on infant and young child feeding, by intervention area and survey round^1^

	Endline 2014 (T1)		Follow-up 2016 (T2)		Change over time (T2 − T1)		
	Intensive (*n* = 1001)	Nonintensive (*n* = 1000)	Intensive (*n* = 1200)	Nonintensive (*n* = 1200)	Intensive (*n* = 2201)	Nonintensive (*n* = 2200)	*P* for DID
Breastfeeding messages							
Ever heard about “Putting baby to the breast immediately after birth”	98.8***^2^	87.5	97.1	96.1	−1.7	8.6^###^^3^	0.0001
Sources of this information^4^							
BRAC health workers	86.9***	18.6	84.1***	23.8	−2.9	4.8^#^	
Other health workers	18.4***	46.6	28.9***	60.5	10.7^#^	14.0^##^	
Husband	1.0***	5.1	0.7*	2.8	−0.3	−2.3	
Mother or mother-in-law	6.2***	18.1	18.5**	34.7	12.3^###^	16.8^##^	
Other family members	7.8***	32.9	24.8***	60.9	17.0^##^	28.0^###^	
Mass media	11.1**	25.6	10.6***	25.3	−0.2	−0.3	
Ever heard about “Giving only colostrum in the first day or two until breastmilk comes in”	97.7***	82.7	96.8***	92.8	−0.9	10.1^##^	0.002
Sources of this information							
BRAC health workers	86.9***	17.1	82.9***	23.4	−4.3	5.8^#^	
Other health workers	17.3***	44.3	28.1***	62.8	11.0^#^	18.5^##^	
Husband	1.1**	4.0	0.8*	2.7	−0.4	−1.3	
Mother or mother-in-law	5.7***	19.7	17.7**	31.8	11.9^###^	12.6^#^	
Other family members	7.8***	32.3	59.6***	57.8	16.1^##^	26.0^###^	
Mass media	10.4***	24.8	10.4***	21.9	0.3	−2.0	
Ever heard about “No prelacteals (honey/mustard oil)”	92.6***	66.7	91.3**	82.2	−1.3	15.5^###^	0.0001
Sources of this information							
BRAC health workers	86.6***	18.6	84.1***	23.5	−2.9	4.6	
Other health workers	17.0***	47.4	26.2***	61.1	9.5	13.9^#^	
Husband	1.0***	4.7	1.1	2.5	0.1	−2.1	
Mother or mother-in-law	5.4***	15.4	15.9**	29.1	10.3^##^	14.0^#^	
Other family members	7.8***	31.9	24.2***	52.2	16.5^##^	21.0^##^	
Mass media	10.5***	25.2	8.3***	22.7	−1.7	−2.5	
Ever heard about “Feeding only breast milk up to 6 mo”	98.7***	87.6	97.4*	95.6	−1.3	8.0^#^	0.003
Sources of this information							
BRAC health workers	87.5***	19.9	86.0***	24.6	−1.7	4.6	
Other health workers	17.2*	45.1	27.4***	63.3	10.3	18.0^##^	
Husband	1.0**	4.3	0.9	2.3	−0.1	−2.0	
Mother or mother-in-law	5.6***	16.0	15.7**	29.6	10.2^##^	14.1^#^	
Other family members	7.1***	35.1	28.2***	55.2	21.2^##^	20.5^##^	
Mass media	10.4***	25.8	10.1***	22.9	−0.1	−2.2	
Ever heard about “Not giving water/liquids up to 6 mo”	95.3***	70.8	91.9*	86.7	−3.4	15.9^##^	0.0001
Sources of this information							
BRAC health workers	88.6***	18.2	85.8***	23.6	−3.1	4.4	
Other health workers	16.1***	45.5	24.7***	60.6	8.8	15.5	
Husband	1.2**	4.2	1.1*	2.8	−0.1	−1.4	
Mother or mother-in-law	4.9***	16.4	14.3**	27.7	9.2^##^	12.0^#^	
Other family members	6.6***	30.7	25.7***	53.5	19.0^##^	23.4^##^	
Mass media	9.5***	25.7	7.8***	21.2	−1.4	−3.8	
Complementary feeding messages							
Ever heard about “Feeding mashed family food after 6 mo”	91.7***	79.9	96.2	95.0	4.5	15.1^###^	0.003
Sources of this information							
BRAC health workers	88.9***	17.8	86.8***	22.7	−2.3	4.6^#^	
Other health workers	13.7***	37.3	19.3***	51.1	5.7	13.9^#^	
Husband	0.8**	6.1	1.4	2.6	0.6	−3.5	
Mother or mother-in-law	6.3***	20.5	17.9***	35.9	11.5^##^	15.5^##^	
Other family members	7.4***	38.1	32.9***	68.7	25.8^##^	30.7^###^	
Mass media	10.0***	25.4	7.3***	22.7	−2.5	−2.6	
Ever heard about “Feeding eggs, meat, fish, and other animal-source foods to children >6 mo”	93.0***	82.3	96.6	95.8	3.6	13.5^##^	0.005
Sources of this information							
BRAC health workers	89.4***	17.7	86.8***	23.1	−2.8	4.9^#^	
Other health workers	13.5***	38.6	18.4***	51.2	4.9	12.7	
Husband	0.6	5.2	1.5	2.8	0.8	−2.4	
Mother or mother-in-law	5.8***	17.7	16.2***	34.6	10.4^##^	17.3^##^	
Other family members	7.5***	38.5	32.6***	68.8	25.3^##^	30.4^###^	
Mass media	10.0***	28.3	8.5	24.6	−1.3	−3.5	
Ever heard about “Cooking children's food with oil or mixing oil in children's food”	58.3***	37.0	68.8	42.4	10.5	5.4	0.585
Sources of this information							
BRAC health workers	87.5***	13.5	88.1***	21.8	0.6	8.4	
Other health workers	14.0***	38.7	13.7***	47.2	−0.4	8.7	
Husband	1.4*	3.0	1.2	3.1	−0.7	0.1	
Mother or mother-in-law	4.3**	14.9	14.2***	22.6	9.5^##^	8.2	
Other family members	6.2***	37.3	21.4***	56.5	14.6^#^	18.5^##^	
Mass media	6.0***	23.5	4.6***	19.7	−1.0	−2.1	

1 Values are percentages. BRAC, Bangladesh Rural Advancement Committee; DID, difference in difference; T, time.

2 Significant difference between intensive and nonintensive areas: *P < 0.05, **P < 0.01, ***P < 0.001.

3 Significant difference between endline 2014 and follow-up 2016: ^#^*P* < 0.05, ^##^*P* < 0.01, ^###^*P* < 0.001. *P* values obtained from regression models controlling for geographic clustering with district as a fixed effect and upazila as a random effect.

4 Among those who ever received information.

The percentage of mothers who shared IYCF messages with other people was higher in intensive than in nonintensive areas in 2014 and increased significantly over time in both areas (**Table 3**). For breastfeeding messages, these changes ranged from 18 to 23 pp in intensive areas and from 32 to 41 pp in nonintensive areas, with changes in messages on early initiation of breastfeeding and feeding colostrum being higher in nonintensive areas. Similar positive changes were also observed for several complementary feeding messages, ranging from 19 to 24 pp in A&T areas and 11 to 37 pp in nonintensive areas. Compared to endline, more mothers shared with female family members in both areas and shared with other women in the community at follow-up. Increased receipt and sharing of IYCF information in both areas at follow-up, particularly higher in nonintensive areas, indicated a wider diffusion of information over time.

**TABLE 3:** Information shared by mothers on infant and young child feeding, by intervention area and survey round^1^

	Endline 2014 (T1)		Follow-up 2016 (T2)		Change over time (T2 − T1)		
	Intensive (*n* = 1001)	Nonintensive (*n* = 1000)	Intensive (*n* = 1200)	Nonintensive (*n* = 1200)	Intensive (*n* = 2201)	Nonintensive (*n* = 2200)	*P* for DID
Breastfeeding messages							
Ever shared about “Putting baby to the breast immediately after birth”	52.2***^2^	28.3	71.2	67.3	19.0^#^^3^	39.0^###^	0.029
To whom mothers shared information^4^							
Husband	12.5	13.1	14.2	6.9	0.6	−6.0	
Mother or mother-in-law	21.7	20.5	29.9**	17.6	6.8	−2.3	
Other family members	40.4**	27.6	58.2	51.4	17.9	24.2^##^	
Other women in community	69.2	60.8	66.0***	78.8	−3.4	16.6^##^	
Other men in community	1.9	1.8	0.6	0.5	−1.3	−1.3	
Ever shared about “Giving only colostrum in the first day or two until breast milk comes in”	50.7***	24.0	68.3	65.2	17.7^#^	41.2^###^	0.019
To whom mothers shared information							
Husband	11.4	12.1	13.1*	5.6	0.5	−6.4^##^	
Mother or mother-in-law	22.5	18.3	27.3*	17.8	3.6	−0.1	
Other family members	39.8**	26.7	55.2	47.8	15.2	21.9^##^	
Other women in community	70.6	63.8	69.6*	78.8	−1.4	14.0^#^	
Other men in community	1.6	1.7	1.1	0.4	−0.5	−1.2	
Ever shared about “No prelacteals (honey/mustard oil)”	45.6***	20.3	63.1*	54.6	17.5^#^	34.3^###^	0.080
To whom mothers shared information							
Husband	9.9	11.8	14.0*	5.3	2.9	−5.8^#^	
Mother or mother-in-law	21.1	17.7	31.3***	14.7	8.8^#^	−3.1	
Other family members	41.9**	28.1	54.6	45.3	12.6	17.1^#^	
Other women in community	70.8	66.0	70.5**	79.9	−0.7	13.1^##^	
Other men in community	1.8	2.5	0.7	0.6	−1.0	−2.1	
Ever shared about “Feeding only breast milk up to 6 mo”	48.7***	25.5	71.7*	62.3	23.0^#^	36.8^###^	0.184
To whom mothers shared information							
Husband	11.7	12.9	12.7*	5.6	0.0	−7.1^#^	
Mother or mother-in-law	21.6	17.7	29.2**	14.1	6.3	−2.9	
Other family members	39.0**	24.7	55.2	48.1	16.5	23.4^##^	
Other women in community	70.6	62.8	72.6	76.8	1.4	12.2^##^	
Other men in community	2.3	2.8	0.9	0.9	−1.0	−2.1	
Ever shared about “Not giving water/liquids up to 6 mo”	46.2***	20.3	64.3*	52.0	18.2	31.7^###^	0.171
To whom mothers shared information							
Husband	11.0	14.3	14.4*	6.3	1.9	−7.7^#^	
Mother or mother-in-law	20.8	16.8	30.7***	13.3	8.4	−3.2	
Other family members	37.9**	23.2	55.4	44.1	17.2	21.0^#^	
Other women in community	74.2	66.0	72.0*	81.3	−2.6	13.1^##^	
Other men in community	2.6	2.5	0.7	1.0	−1.6	−1.6	
Complementary messages							
Ever shared about “Feeding mashed family food after 6 mo”	46.6***	25.5	70.8	61.8	24.2^#^	36.3^###^	0.190
To whom mothers shared information							
Husband	13.1	15.3	12.6	8.0	−1.8	−6.9^#^	
Mother or mother-in-law	19.5	19.2	30.4**	14.4	9.5	−3.9	
Other family members	38.8**	25.5	56.0	47.5	17.5	22.4	
Other women in community	72.3	66.7	71.7*	80.0	−0.7	12.5^#^	
Other men in community	2.2	2.0	1.2	0.3	−0.6	−1.8	
Ever heard about “Feeding eggs, meat, fish, and other animal source foods to children >6 mo”	48.2***	26.7	70.3	63.2	22.1^#^	36.5^###^	0.129
To whom mothers shared information							
Husband	14.5	16.9	13.1	8.6	−2.8	−8.2^##^	
Mother or mother-in-law	19.7	19.1	29.3**	14.3	8.1	−4.3	
Other family members	38.0**	27.0	57.4	49.2	20.5	23.2^#^	
Other women in community	72.4**	64.8	71.7**	79.4	−1.4	13.6^##^	
Other men in community	2.3	1.1	1.4	0.8	−0.6	−0.5	
Ever heard about “Cooking children's food with oil or mixing oil in children's food”	26.8***	11.2	45.8**	22.2	19.0^#^	11.0^#^	0.352
To whom mothers shared information							
Husband	13.8	14.3	14.9	8.3	0.3	−7.1	
Mother or mother-in-law	17.9	29.5	35.5**	16.5	16.0^##^	−12.2	
Other family members	36.6**	19.6	56.1	50.8	18.9	29.1^##^	
Other women in community	74.6*	58.0	70.5*	79.7	−4.5	22.0^#^	
Other men in community	3.7*	0.0	0.7	0.4	−3.4	0.3	

1 Values are percentages. DID, difference in difference; T, time.

2 Significant difference between intensive and nonintensive areas: *P < 0.05, **P < 0.01, ***P < 0.001.

3 Significant difference between endline 2014 and follow-up 2016: ^#^*P* < 0.05, ^##^*P* < 0.01, ^###^*P* < 0.001. *P* values obtained from regression models controlling for geographic clustering with district as a fixed effect and upazila as a random effect.

4 Among those who ever shared information.

### Social norms about IYCF

Perceptions of descriptive norms related to IYCF were generally higher in intensive areas (60–80%), with additional changes over time (8–16 pp) (**Table 4**). In nonintensive areas, there were higher perceptions of descriptive norms for both breastfeeding (13–17 pp) and complementary feeding (27–28 pp) between endline and follow-up. The changes in perceptions of descriptive norms related to complementary feeding were higher in nonintensive compared to intensive areas. Perception of injunctive norms was generally high in both areas.

**TABLE 4:** Mother's perception of social norms related to IYCF practices, by intervention area at follow-up^1^

	Endline 2014 (T1)		Follow-up 2016 (T2)		Change over time (T2 − T1)		
	Intensive (*n* = 1001)	Nonintensive (*n* = 1000)	Intensive (*n* = 1200)	Nonintensive (*n* = 1200)	Intensive (*n* = 2201)	Nonintensive (*n* = 2200)	*P* for DID
Descriptive norms: Proportion of mothers who believe most mothers in their community do the following							
Early initiation of breastfeeding							
Breastfeed immediately after delivery	82.3**^2^	67.3	90.6**	83.9	8.3^##^^3^	16.6^##^	0.137
Do not give prelacteals to babies right after delivery	56.9***	30.2	73.0***	46.7	16.1^#^	16.5^##^	0.960
Exclusive breastfeeding							
Breastfeed the child exclusively for 6 mo	68.4***	44.8	70.9**	57.3	2.5	12.5^#^	0.248
Complementary feeding							
Feeding mashed family food after 6 mo	74.7***	57.6	89.4	85.8	14.7^##^	28.2^###^	0.031
Feeding eggs, meat, fish, and other animal-source foods to children aged > 6 mo	67.7***	40.4	75.5	67.5	7.8	27.1^##^	0.010
Injunctive norms: Proportion of mothers who strongly agree with the following statements: Most people who are important to me …							
Early initiation of breastfeeding							
Think that a mother, after normal delivery, can breastfeed her infant within 1 h	—	—	98.8	98.8	—	—	
Think that a mother after cesarean delivery can breastfeed her infant within 1 h	—	—	87.3***	58.9	—	—	
Exclusive breastfeeding					—	—	
Think that I should feed my infant only breast milk, and no other food, water, or infant formula for the first 6 mo	—	—	90.4*	84.8	—	—	
Do not approve of me giving my baby water before she/he reaches age 6 mo	—	—	75.5*	66.2	—	—	
Do not approve of me giving my baby infant formula before she/he reaches 6 mo	—	—	83.4	75.7	—	—	
Do not approve of me giving my baby semisolid or solid foods before age 6 mo	—	—	87.2	81.5	—	—	
Complementary feeding							
Think that I should add egg or fish or liver or meat or chicken in addition to other foods every day, starting at 7 mo	—	—	97.8	98.1	—	—	
Think that I should feed my baby mashed family cooked foods (rice, vegetable, daal) along with breast milk after age 6 mo	—	—	98.0	98.5	—	—	
Approve of me feeding my child after 6 mo at least 3 meals/d	—	—	94.1	96.6	—	—	

1 Values are percentages. DID, difference in difference; IYCF, infant and young child feeding; T, time.

2 Significant difference between intensive and nonintensive areas: *P < 0.05, **P < 0.01, ***P < 0.001.

3 Significant difference between endline 2014 and follow-up 2016: ^#^*P* < 0.05, ^##^*P* < 0.01, ^###^*P* < 0.001. *P* values obtained from regression models controlling for geographic clustering with district as a fixed effect and upazila as a random effect.

### Association of networks of known adopters, diffusion of information, and social norms with IYCF practices

At follow-up, compared to mothers in the lowest quartile of social network score, mothers in the third and fourth quartiles were 1.51–2.68 times more likely to have optimal IYCF (**Table 5**). Similar results were observed for received information score, shared information score, and social norms for early initiation of breastfeeding and exclusive breastfeeding, and for shared information score for minimum dietary diversity.

**TABLE 5:** Association of IYCF practices with scores at follow-up on social networks of known adopters, diffusion of IYCF information, and mothers’ perceived social norms^1^

	Early initiation of breastfeeding (*n* = 2400)	Exclusive breastfeeding (*n* = 1202)	Minimum dietary diversity (*n* = 1198)
Social network score of known adopters			
Quartile 1	Ref	Ref	Ref
Quartile 2	1.16 (0.96, 1.41)	1.11 (0.69, 1.78)	1.11 (0.74, 1.67)
Quartile 3	1.51**^2^ (1.11, 2.07)	1.92** (1.30, 2.83)	1.83** (1.24, 2.71)
Quartile 4	2.17*** (1.55, 3.05)	2.68*** (1.57, 4.55)	2.15** (1.39, 3.32)
Received information score			
Quartile 1	Ref	Ref	Ref
Quartile 2	1.31* (1.06, 1.62)	1.26 (0.86, 1.85)	0.81 (0.58, 1.13)
Quartile 3	1.89*** (1.39, 2.57)	1.48* (1.02, 2.15)	1.34 (0.90, 2.00)
Quartile 4	2.30** (1.39, 3.82)	2.38*** (1.58, 3.60)	1.46 (0.92, 2.32)
Shared information score			
Quartile 1	Ref	Ref	Ref
Quartile 2	0.99 (0.77, 1.27)	1.19 (0.80, 1.78)	0.98 (0.73, 1.32)
Quartile 3	1.32^+^ (0.98, 1.78)	1.58* (1.04, 2.42)	1.36^+^ (0.95, 1.96)
Quartile 4	1.91** (1.28, 2.84)	1.59^+^ (0.95, 2.65)	1.54^+^ (0.96, 2.47)
Descriptive social norm score			
Quartile 1	Ref	Ref	Ref
Quartile 2	1.31* (1.04, 1.65)	1.17 (0.90, 1.51)	1.08 (0.83, 1.42)
Quartile 3	1.54** (1.15, 2.07)	0.96 (0.63, 1.47)	1.16 (0.70, 1.92)
Quartile 4	1.79** (1.17, 2.75)	2.56** (1.45, 4.52)	1.39 (0.88, 2.19)
Injunctive social norm score			
Quartile 1	Ref	Ref	Ref
Quartile 2	1.28^+^ (0.94, 1.75)	1.65** (1.14, 2.40)	0.92 (0.65, 1.31)
Quartile 3	1.23 (0.94, 1.61)	2.14*** (1.47, 3.13)	1.00 (0.69, 1.45)
Quartile 4	1.92*** (1.34, 2.73)	4.38*** (2.86, 6.70)	0.99 (0.64, 1.54)

1 Values are ORs (95% CIs). Models adjusted for social economic status, maternal education, parity, child age, sex, and geographic clustering with district as a fixed effect and upazila as a random effect. Each factor of diffusion, network, and social norms was run in a separate model. IYCF, infant and young child feeding; Ref, reference.

2 *, **, ***, ^+^Different from reference group: ^+^*P* < 0.1, *P < 0.05, **P < 0.01, ***P < 0.001.

### Mediation by networks of known adopters, diffusion of information, and social norms

**FIGURE 3:**
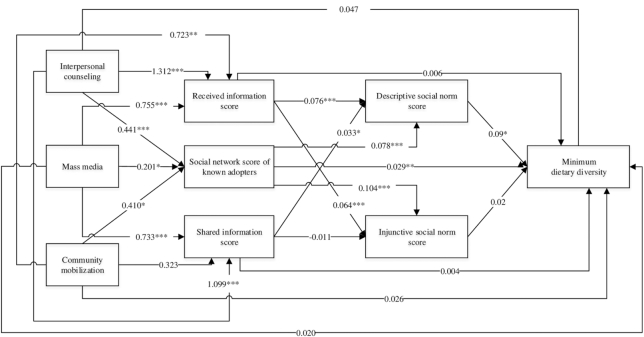
Path analysis for minimum dietary diversity. Significantly different: *P < 0.05, **P < 0.01, ***P < 0.001.

In the path analyses for minimum dietary diversity, mothers exposed to 3 components of the interventions had higher scores for social networks of adopters (β = 0.20–0.44), receiving information (β = 0.72–1.31), and sharing information (β = 0.32–1.10) (**Figure 3**). The social network of adopters and diffusion of information, in turn, were positively associated with both descriptive norms (β = 0.03–0.08) and injunctive norms (β = 0.06–0.10), which were associated with higher minimum dietary diversity (β = 0.02–0.09). The indirect effects, obtained by adding the products of the regression coefficients for each path, show that, for minimum dietary diversity, 34%, 42%, and 43% of the total effects of IPC, MM, and CM, respectively, were explained by improved social networks, diffusion of information, and social norms. The indirect effects for IPC were 61% for early initiation of breastfeeding and 39% for EBF; the indirect effect of MM was 78% for EBF (**Supplemental Figures 1** and **2**).

## Discussion

Large-scale behavior change communication interventions in which intensive IPC has been combined with MM and CM activities have had positive impacts on IYCF practices in several countries (20, 21, 27, 28). The current study advances previous knowledge by examining the paths connecting from interventions to the IYCF practices. Two years after termination of the initial donor's support, mothers’ networks of known adopters, receiving and sharing of IYCF information, and social norms remained high in intensive areas and increased substantially in nonintensive areas. Differences in social networks, diffusion of information, and social norms, in turn, partially explained the intervention differences in IYCF practices that were previously reported (20, 21). These paths explained 34–78% of the total effects.

Two years after endline, given BRAC's continued implementation of IYCF interventions as part of its routine Essential Health Care program services (25), mothers’ awareness of IYCF messages was maintained in intensive areas, and BRAC frontline health workers continued to be a major source of IYCF information in those areas; these findings are supported by a recent study of the sustained impacts and continued intervention exposure from the original program (29). In addition, information receiving and sharing increased substantially in nonintensive areas, with the change even larger than in intensive areas for several messages, as expected from hypothesis 1. This change was likely attributed to uptake of A&T materials including tools, materials, and information in nonintensive areas. Furthermore, the government of Bangladesh adopted the A&T MM materials and started broadcasting the IYCF videos again nationally in early 2016. Evidence of a wider diffusion of information beyond BRAC health workers was also shown through increased IYCF information received through other sources (other health workers and family members) in both areas and increased sharing of IYCF information by mothers particularly with other women in their family and in the community. Diffusion of IYCF information in the community was corroborated by a larger network of known adopters. Our findings are in line with a recent nutrition education intervention in Bangladesh showing that mothers passed IYCF information on to neighboring nonparticipants and the neighbors scored higher on IYCF knowledge, indicating an information spillover effect (15).

As expected from hypothesis 2, networks of known adopters, receiving and sharing IYCF information, and social norms were strongly related with breastfeeding practices. The total impact of nutrition social and behavior change communication activities on breastfeeding practices depends not only on intervention participants’ acceptance of promoted behaviors, but also on the extent to which the primary recipients of the interventions spread the information to key influential members of the general population. In the context of rural Bangladesh where collectivism (such as being a member of a family, extended family, or extended relations in a community group) is common (30), the role of social norms on breastfeeding practice is particularly important to consider (18). The A&T intervention was successful both in disseminating information to primary target audiences and in mobilizing social networks to bring about large-scale diffusion of information. During the intensive intervention period (2010–2014), A&T used a combination of strategies, including interpersonal communication, CM, a multimedia campaign, and engagement in dialogue with national, district, and community leaders. The CM activities involved religious leaders, informal healthcare providers (village doctors), traditional birth attendants, government health and family welfare staff, schoolteachers, members of village health committees, fathers, and grandmothers in each family. By bringing together various influential actors, community forums raised greater awareness of appropriate IYCF practices.

Although we found a strong association between social networks and sharing information with minimum dietary diversity, the role of social norms is less apparent. Different from breastfeeding practices that mainly depend on social and behavioral determinants, complementary feeding practices also required availability of and access to food and resources. Access to food, especially high-quality foods, is limited in rural Bangladesh, and lack of resources (24) and restricted women's agency have been identified as salient barriers to trying and adopting recommended complementary feeding practices even when caregivers have high self-efficacy for following recommended complementary feeding practices (31). Additional efforts to address factors related to food security and women's agency, therefore, are critical for improving complementary feeding practices.

Our results are consistent with the conceptual framework and hypothesis 3 that a portion of the effect of exposure to the messages from the 3 intervention components was received and shared in mothers’ social networks, which in turn affected social norms and then practices. A similar effect has been studied in another type of intervention, where conversations about the warnings were a key mechanism through which pictorial warnings influenced smoking quit attempts (13). These results provide evidence to support that messages from interventions (e.g., IPC) and campaigns (e.g., MM or social mobilization) are filtered through social networks (12), which then spread information and translate messages into norms (13, 14), leading to changes in practices. The current study only measured social norms as reported by mothers and not by other members of their social network. Additional measures of large and complex social networks would provide more comprehensive insight on how information and norms spread in the community.

Using 2 rounds of household surveys at endline and follow-up, we analyzed rich data at both individual and community levels on several dimensions related to information received and shared, sources of information, networks of known adopters, and perceived individual social norms (both descriptive and injunctive norms). Most information was collected at multiple time points, allowing us to compare the patterns of changes over time. Our results lend evidence to findings in a previous paper (22) where increased IYCF knowledge and practices were observed in nonintensive areas despite low exposure to BRAC frontline health workers, which may have resulted from the positive shifts in social factors. Thus, this present study goes beyond the impact analyses to further examine the complex relation between caregivers and their social environments, in particular focusing on diffusion of IYCF information through social networks to shift social norms related to IYCF.

Although we acknowledge the important influence of social networks on IYCF behaviors, we did not measure all dimensions of social networks. Our measure mainly focused on the proportion of mothers with known adopters and the size of the network (numbers of adopters), but we did not distinguish other dimensions such as direct and indirect ties, or the closeness, betweenness, and power (32) or conduct a social network analysis. Further in-depth study may provide a more complete architecture of social networks operating among caregivers within their communities and their influences on IYCF practices. Our measures of IYCF practices, information diffusion, networks, and social norms were based on self-reports, which may be influenced by recall bias and social desirability bias, although standard research methods for reducing bias were followed. We addressed this issue by assessing the role of social desirability (33) in relation to IYCF practices and did not find evidence of reporting bias influenced by respondents’ desire for social approval (20, 21).

In conclusion, IYCF is a complex set of behaviors, influenced by caregivers and their social environment. Until age-appropriate IYCF practices are normalized in society with wide diffusion and acceptance, continued delivery of quality counseling and multichannel behavior change interventions need to be maintained. Diffusion of IYCF information in the community through social networks, reinforced by positive social norms for specific messages promoted over time, will contribute to positive changes in IYCF practices that may be achieved and sustained through large-scale social and behavior change interventions.

## Supplementary Material

nxz167_Supplemental_File

## Notes

Supported by the Bill & Melinda Gates Foundation, through Alive & Thrive, managed by FHI 360; additional financial support for the evaluation study was provided by the CGIAR Research Program on Agriculture for Nutrition and Health (A4NH), led by the International Food Policy Research Institute.

Author disclosures: PHN, EAF, SSK, AAZ, AJ, LMT, TS, and PM, no conflicts of interest. TS was a member of the intervention implementation team that designed and implemented the interventions studied in this manuscript. She reviewed the manuscript and provided interpretation of the results, but final decisions for content lay with the primary authors from the evaluation team (PHN, EAF, SSK, AAZ, AJ, LMT, and PM).

Supplemental Table 1 and Supplemental Figures 1 and 2 are available from the “Supplementary data” link in the online posting of the article and from the same link in the online table of contents at https://academic.oup.com/jn/.

Abbreviations used: A&T, Alive & Thrive; BRAC, Bangladesh Rural Advancement Committee; CM, community mobilization; EBF, exclusive breastfeeding; IPC, interpersonal counseling; IYCF, infant and young child feeding; MM, mass media; pp, percentage point(s).
