# Supplementary material for: Information Diffusion and Social Norms Are Associated with Infant and Young Child Feeding Practices in Bangladesh
Source: J Nutr. 2019 Aug 8;149(11):2034–45. doi: 10.1093/jn/nxz167 (PMC6825823; doi:10.1093/jn/nxz167)

Supplementary data

Supplemental Table 1. Description of differences between intervention groups in Bangladesh at endline.

**Intervention component**	**Non-intensive**	***Intensive***
Interpersonal counseling	Standard care and nutrition counseling by BRAC health volunteers, *Shasthya Sebika* (SS) who are assigned 250-300 households in their community; expected to visit households monthly, provide health products mainly related to maternal and child health, give messages for 10 key health topics including breastfeeding and immunization, refer for child illnesses, help identify pregnancies and link women to antenatal care, be present at or soon after a delivery.<br><br>BRAC health workers, *Shasthya Kormi* (SK) (one per 2500-3000 households) provide antenatal care and postnatal care to mothers who choose to use BRAC's services and motivate mothers to deliver at a health facility.	Standard care (as described in non-intensive), plus added *intensified* counseling:<br>- Additional emphasis on early initiation, no pre-lacteals and EBF, animal source foods, feeding quantity during ANC and PNC services provided by the SK<br>- Facilitate initiation of BF within one hour by incentivized BRAC health volunteer<br>- Incentivized counseling on IYCF by SS during monthly door-to-door visits to all households with children under two years of age<br>- Counseling by full-time dedicated IYCF worker, *Pushti Kormi*, (PK) (one per 2000-2500 households) to address difficulties, complete volunteer records of home visits, train mothers at home on complementary feeding at six months, and ensure the following schedule of contacts with mothers during the first two years: monthly from 0 to 8 months, every other month to 12 months, one more visit each between 15-18 and 23-24 months<br>- Additional emphasis given to IYCF at community meetings facilitated by BRAC's ANC/PNC provider in the community with pregnant and recently delivered women
Mass media	TV and radio Spots: 7 IYCF focused TV commercials aired via four most watched (by rural mothers) national channels and programs 6 to 12 times a day on three days of the week; and radio stories based on TVCs aired on four radio stations and news/sports programs targeted to male audiences	- *Same as non-intensive*<br>- Added screening of same TV spots plus a cartoon film on IYCF (Meena) via community-based video shows in areas where TV reach was lower ("media dark" strategies) accompanied by community dialogue and quiz shows
	1. Early Initiation of Breastfeeding: 42''<br>https://www.aliveandthrive.org/resources/tv-spot-early-initiation-of-breastfeeding-bangladesh/<br>Insufficient Milk: 62''<br>https://www.aliveandthrive.org/resources/tv-spot-perception-of-insufficient-milk-bangladesh/<br>Father's Involvement: 43''<br>https://www.aliveandthrive.org/resources/tv-spot-fathers-involvement-bangladesh/<br>Animal Food: 42''<br>https://www.aliveandthrive.org/resources/tv-spot-animal-food-bangladesh/<br>Feeding Quantity" 63''<br>https://www.aliveandthrive.org/resources/tv-spot-feeding-quantity-bangladesh/<br>Poor Appetite: 42''<br>https://www.aliveandthrive.org/resources/tv-spot-poor-appetite-bangladesh/<br>IYCF plus handwashing: 20''<br>https://www.aliveandthrive.org/resources/tv-spot-iycf-plus-handwashing-bangladesh/	
Community mobilization	Basic community mobilization through local meetings on topics such as family planning, pregnancy registration, antenatal care	-Local meetings organized by BRAC managers to introduce the A&T program and activities of workers, targeted to school teachers, adolescents, religious leaders, village doctors, district hospital/clinic doctors, community elites on the topic of nutrition and IYCF; fathers' meetings added with emphasis on handwashing linked to complementary feeding<br>-Village theater shows for the general public to generate discussion about the importance of nutrition and IYCF; role of worker

Supplementary data

Supplemental Figure 1. Path analysis for early initiation of breastfeeding.

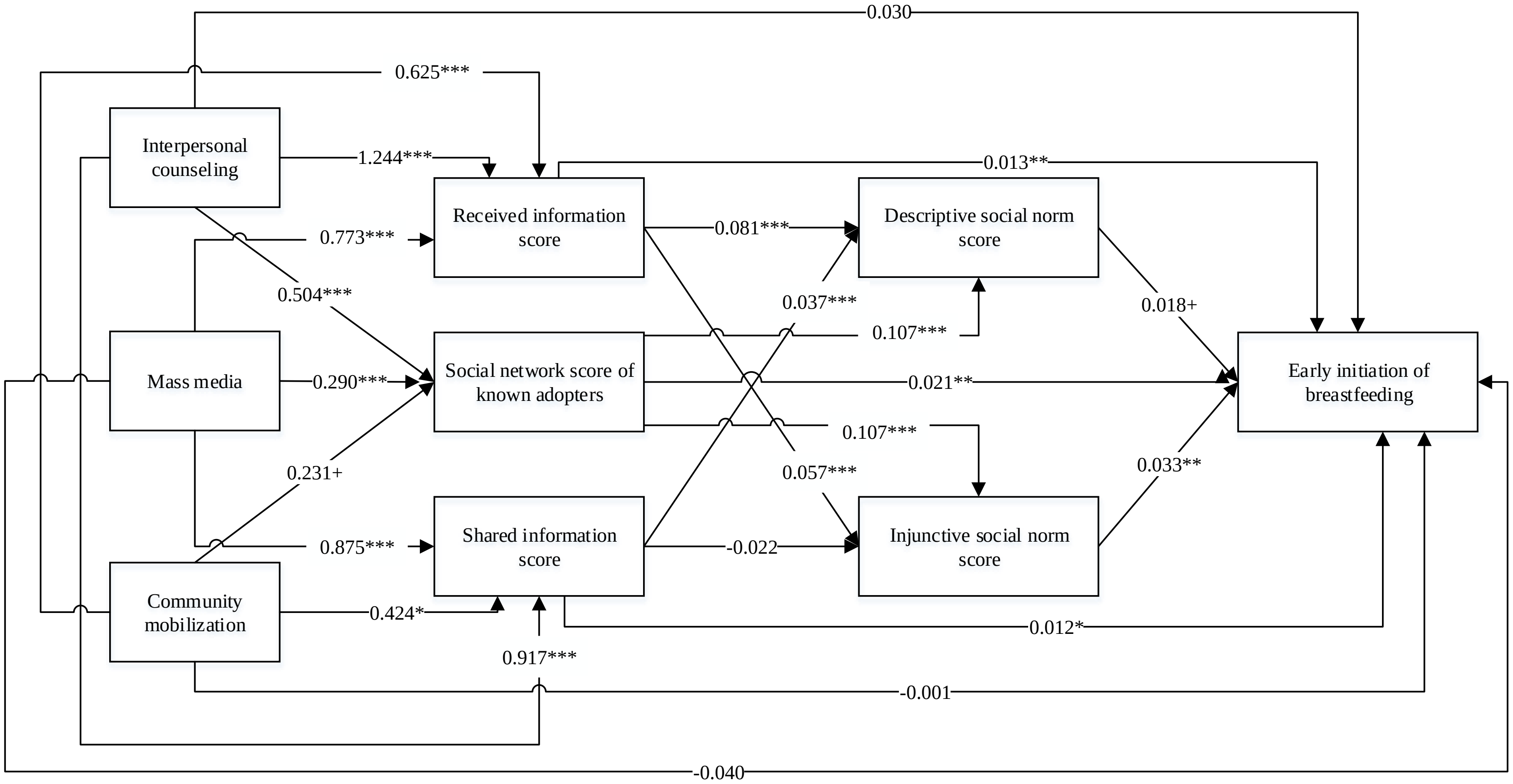

Supplementary data

Supplemental Figure 2. Path analysis for exclusive breastfeeding.

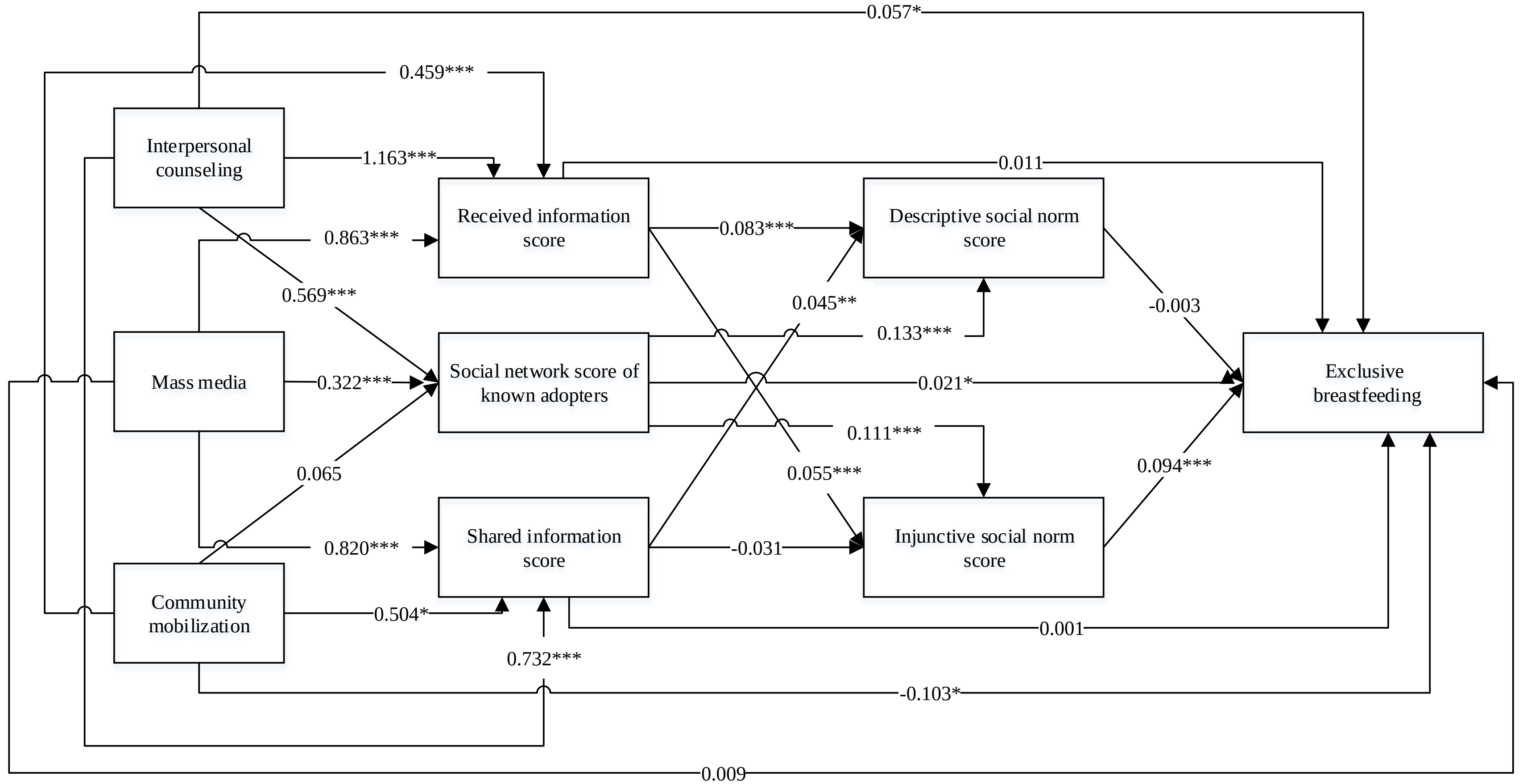

Supplement: nxz167_Supplemental_File [file nxz167_supplemental_file.pdf]
